# Risk of postoperative complications among ulcerative colitis patients treated preoperatively with vedolizumab: a matched case-control study

**DOI:** 10.1186/s12893-020-00698-8

**Published:** 2020-03-05

**Authors:** Jeong Yeon Kim, Karen Zaghiyan, Amy Lightner, Phillip Fleshner

**Affiliations:** 1 Department of Surgery, Hallym University College of Medicine, Dongtan, South Korea; 2 Division of Colorectal Surgery, Cedars-Sinai Medical Center, 7 Beverly Blvd., Suite 101, Los Angeles, California 90048 USA; 3 Department of Colon and Rectal Surgery, Cleveland Clinic Foundation, Cleveland, Ohio USA

**Keywords:** Ulcerative colitis, Vedolizumab, Postoperative complications

## Abstract

**Background:**

Although biologic agents have revolutionized the medical management of severe ulcerative colitis (UC), there is considerable controversy regarding adverse effects of vedolizumab on surgical outcomes. We evaluated 30-day postoperative morbidity in UC patients undergoing abdominal colectomy (AC) treated with vedolizumab before surgery.

**Methods:**

From 2007 to 2017, 285 patients were enrolled in prospectively maintained database evaluating the role of clinical, serologic markers with clinical phenotypes in UC. The patients treated with vedolizumab within 12 weeks of AC was queried, then matched 1:3:3 into 3 preoperative treatment groups based on age, gender and surgical treatment of UC; ileal pouch-anal anastomosis (IPAA) with ileostomy vs total colectomy with end stoma: a) vedolizumab (*n* = 25); b) anti-tumor necrosis factor (anti-TNF) (*n* = 74); and c) no biologics (*n* = 54). Thirty-day postoperative complications among patient groups were compared.

**Results:**

The 3 patient groups were well-matched in other characteristics including disease duration, disease extent, medication history and preoperative serological data. There were no significant differences in the overall incidence of postoperative complications among patients treated preoperatively with vedolizumab, anti-TNFs, or no biologics (44% vs. 45% vs. 37%; *p* = 0.67). Although there was no significant difference between patient cohorts in infectious complications (*p* = 0.20), postoperative ileus (POI) was significantly more common among the vedolizumab group (*n* = 9; 36%) compared to anti-TNF (*n* = 12; 16%) or no biologics (*n* = 5; 9%) (*p* = 0.01). Multivariable analysis showed that vedolizumab treatment prior to surgery was an independent risk factor for POI (OR: 5.16, 95% CI; 1.71–15.52; *p* = .004).

**Conclusion:**

Although preoperative vedolizumab exposure did not influence the rate of overall 30-day postoperative complications, vedolizumab tends to increase incidence of POI.

## Background

By targeting various host immune molecules such as tumor necrosis factor (TNF), interleukins and adhesion molecules present on the surfaces of lymphocytes, biologics agents have revolutionized the medical management of inflammatory bowel disease (IBD). Despite these improvements, the need for surgical intervention in IBD remains high [1, 2], predisposing many patients to surgical intervention during exposure to biologic therapy.

Vedolizumab is a gut specific humanized monoclonal antibody which selectively binds to the α4β7 integrin, thereby blocking its interaction with mucosal addressin cell adhesion molecule-1 (MAdCAM-1). This action inhibits the migration of lymphocytes into the gastrointestinal lymph tissue and arrests the chronic inflammatory state of both ulcerative colitis (UC) and Crohn’s disease (CD). This mechanism of action is a potential concern for surgeons as leukocyte migration to the intestinal mucosa may be vital for bowel inflammation [3–5].

The literature regarding potential effects of vedolizumab on surgical outcomes in IBD is conflicting, with some studies suggesting no effect [6–8] and others showing significant associations with surgical site infection [9–11]. These inconsistent findings may be attributed to several factors including retrospective study design, single institution experiences, variable duration of biologic therapies and time periods between the last biologic dose and surgery, heterogeneous patient populations and variable combinations of CD and UC [12]. In an effort to address some of these study concerns, we performed a matched case-control study of postoperative morbidity in medically refractory UC patients treated with vedolizumab before surgery. We hypothesized that postoperative complications would be comparable across vedolizumab treated, anti-TNF treated and no biologic patient groups.

## Methods

### Study population

A prospectively maintained database from single tertiary hospital, evaluating the role of clinical, serologic and genetic markers with clinical phenotypes in IBD was queried for data on affected individuals with medically refractory UC undergoing first abdominal major surgery between January 2007 and June 2017. Patients requiring surgery for chronic disease with cancer, CD, indeterminate colitis (IC), or those treated with ustekinumab prior to surgery were excluded. Those individuals with documented preoperative exposure to more than three times of vedolizumab within 12 weeks prior to surgery were selected and then matched 3:3:1 into 3 preoperative treatment groups (no biologics, anti-TNF treated, vedolizumab treated) based on age, gender and surgical procedure (abdominal colectomy with Brooke ileostomy or abdominal colectomy with ileal pouch-anal anastomosis (IPAA) with loop ileostomy). Approval for all research-related activities was obtained from the Institutional Review Board of the Cedars-Sinai Medical Center (#33934).

### Assessment of clinical characteristics and complications

Demographic information and characteristics of the disease and its treatment were tabulated. Patient factors included gender, age at surgery, body mass index (BMI), preoperative weight loss, smoking history, disease duration, disease extent, disease activity, medication history and number of stages to complete an IPAA. Preoperative weight loss was defined as greater than 10% loss of total body weight observed within 3 months of surgery. Disease duration was defined as the time interval between the diagnosis of UC and surgery. Smoking was defined as patients who smoked at the time of surgery or who had quit smoking before colectomy. We descripted disease extent, disease severity based on Montreal classification [13]. Anatomical extent of the disease was postoperatively categorized as left-sided colitis(E2) or pancolitis(E3). Disease severity was preoperatively categorized as moderate UC or severe UC. Disease activity was divided into two groups; acute vs chronic. Acute disease was defined as an acute condition needing emergency operation such as perforation or megacolon. Chronic activity was defined as a chronic disease condition needing elective surgery. Medication history included ever use of oral or parenteral corticosteroids or 6-mercaptopurine (or azathioprine) /methotrexate (6-MP / MTX) prior to surgery. Preoperative lab values (white blood cell count, platelet count and albumin) within 30 days of surgery were tabulated as surrogates of the patient’s preoperative surgical risk. We followed guideline for peri, post-operative care by Enhanced Recovery After surgery (ERAS) Society Recommendation [14].

Postoperative complications were recorded within 30 days of the index operation. The occurrence of postoperative complications (infection, ileus and other sequelae) were identified from the medical files of all patients. All patients were systematically evaluated by the surgeon 7, 30 days after surgery in outpatient’s clinic. The complications were categorized as infection, postoperative ileus (POI), other morbidity and readmission. Infectious complications included surgical site infections (wound infection, abdominal/pelvic abscess), urinary tract infection and pneumonia. All patients were offered oral intake on postoperative day 1. POI was defined clinically as nausea, vomiting, or abdominal distention requiring regression or cessation of diet, placement of nasogastric tube, or intravenous nutrition. The Grade I-POI was defined as POI without a clear mechanical obstruction identified radiologically. Grade II-POI was diagnosed with similar symptoms to Grade I-POI with a definite transition zone seen on imaging or at surgery.

### Statistical analysis

Summary statistics were represented by counts (percentage) or median (range), where appropriate. Categorical variables were compared using a Chi-square test. Comparisons between population medians were performed using a Kruskal Wallis test for nonnormally distributed variables and ANOVA test for normally distributed variables. Chi-square test was used to compare 30-day postoperative complication among three groups (no biologics vs. anti-TNF vs. vedolizumab). Logistic regression model was used to further investigate the association between surgical outcomes and clinical factors. All statistical analyses were performed using SPSS for Windows, version 17.0 (SPSS Inc., Chicago, IL, USA). All reported *p* values were two-sided, with statistical significance set a priori at *p* < 0.05.

## Results

Over the 11-year period, 285 patients had an abdominal colectomy by one surgeon. Total cohort data was tabulated (Table 1). A total of 25 procedures were identified as meeting entry criteria with exposure to vedolizumab. We used 1:3 matching based on age, gender and number of stages of patients treated with vedolizumab, of 77 patients with non-biologics, only 54 patients were eligible for matching condition. Seventy-four controls unexposed to vedolizumab yet who received anti-TNF therapy were matched 1:3. Anti-TNF agents used included infliximab (*n* = 48), adalimumab (*n* = 26). Although there were no significant statistical differences in clinical characteristics between the three groups, age was relatively older in non-biologics (37 vs 32 vs 31, *p* = 0.13), and previous medication of 6-MP or methotrexate rate was relatively less in the non-biologic group than the other two groups (45% vs 84% vs 80%, *p* = 0.001). As expected from strict case-control matching, baseline clinical characteristics were comparable between study groups. (Table 2).

**Table 1:** Patients’ Characteristics

	*N* = 285	%
Gender		
*Male*	141	50.5
*Female*	144	49.5
Age at surgery(Median)	25(17–37)	
BMI(kg/m2) (Median)	22.6(19–27)	
Disease duration (months)	48(23.7–120)	
Smoking		
*Never*	222	77.9
*Prior*	56	19.6
*Current*	7	2.5
Disease extent		
*E2 Left-side colitis*	35	12.3
*E3 Pancolitis*	250	87.7
Disease Severity		
*S2 Moderate UC*	199	69.8
*S3 Severe UC*	86	30.2
Disease activity		
*Acute*	18	93.7
*chronic*	267	6.3
Medication history		
*Steroid*	90	31.6
*6-MP or MTX*	196	68.8
Immunosuppressant		
*Non-biologics*	77	27
*Anti-TNF*	183	64.2
*Anti-TNF adding Vedolizumab*	25	8.8
Operation type		
*Laparoscopy*	209	73.3
*Open*	76	26.7
Operation performed		
*Total abdominal colectomy* *+end stoma*	92	32.3
*Protectomy + IPAA with ileostomy*	193	67.7
Preoperative labs		
*Leukocyte(× 10*^*3*^*/uL) (Median)*	9.4(6.6–12.5)	
*Platelet(×10*^*3*^*/uL) (Median)*	350(274–475)	
*Albumin(g/dl) (Median)*	3.7(3.3–4.0)	

All values expressed as median (range) or n (%)

*BMI* body mass index, *6-MP* 6-mercaptopurine, *MTX* methotrexate, *IPAA* ileal pouch-anal anastomosis, *TNF* tumor necrosis factor

**Table 2:** Clinical Features of the Three Study Cohorts with Case Matched

	None (*n* = 54)	Anti-TNF (*n* = 74)	Vedolizumab (*n* = 25)	p
Gender (M/F)	29:25	35:39	13:12	0.76
Age at surgery (years)	35 (23–49)	30 (21–45)	31 (22–47)	0.31
BMI (kg/m^2^)	23.3(19–28)	22.7(19–26)	21.1(19–26)	0.15
Preoperative weight loss (> 10 lbs) ^*a*^	17 (35)	23 (37)	10 (43)	0.79
*Missing*	6(11)	11(15)	2(8)	0.63
Disease duration (mos)	61 (24–152)	53 (20–195)	60 (24–148)	0.46
Smoking				0.74
*Never*	43 (80)	59 (80)	21 (84)	
*Prior*	8 (15)	13 (17)	4 (16)	
*Current*	3 (5)	2 (3)	0	
Disease extent				0.89
*E2 Left-sided colitis*	7 (13)	9 (12)	4 (16)	
*E3 Pancolitis*	47 (87)	65 (88)	21 (84)	
Disease activity				0.714
*Acute*	3(5.6)	2(2.7)	1(4.0)	
*Chronic*	51(94.4)	73(2.7)	24(4.0)	
Medication history				
*Steroids*	34 (68)	48 (68)	18 (72)	0.92
*6-MP or MTX*	24 (45)	62 (84)	20 (80)	0.001
Operation type				
*open*	16(30)	*19(26)*	*6(16)*	0.83
*laparoscopy*	38(70)	*55(74)*	*19(76)*	
Operation performed				
*Total abdominal colectomy* *+end stoma*	15 (28)	15 (20)	5 (20)	0.57
*Protectomy + IPAA with ileostomy*	39 (72)	59 (80)	20 (80)	
*Ileostomy repair*	46(85)	60(81)	19(76)	0.61
Preoperative labs				
*Leukocyte (×10*^*3*^*/uL)*	8.9 (6.5–12.4)	9 (6.4–13)	9.8 (6.9–12)	0.79
*Platelet (×10*^*3*^*/uL)*	360 (281–475)	350 (280–459)	340 (274–487)	
*Albumin (g/dL)*	3.6 (3.3–4.0)	3.6 (3.2–4.1)	3.9 (3.6–4.2)	0.87 0.25

All values expressed as median (range) or n (%)

*BMI* body mass index, *6-MP* 6-mercaptopurine, *MTX* methotrexate, *IPAA* ileal pouch-anal anastomosis, *TNF* tumor necrosis factor

^a^19 cases with data missing

Postoperative outcomes are shown in Table 3. Complications occurred in 64 patients (42%). Infection (*n* = 11; 20%) and postoperative ileus (*n* = 21; 13.7%) were the most common complications. Other morbidity included small bowel obstruction (SBO) (*n* = 7), dehydration (*n* = 4), and bleeding, *Clostridium difficile* enteritis, unexplained tachycardia, urinary retention, pancreatitis, deep vein thrombosis, stoma pain and rectal stump leak (all *n* = 1). No patient required reoperation in the immediate postoperative period. The overall incidence of postoperative complications was not significantly different among patients treated preoperatively with vedolizumab, anti-TNFs, or no biologic (*p* = 0.67). Although univariate analysis showed no significant difference between patient cohorts in infectious complications, Grade I-POI was significantly more common among patients treated with vedolizumab compared to patients treated with anti-TNF or no biologics (*p* = 0.01). Grade II-POI showed no significant difference between patients cohots (*p* = 0.89). Length of postoperative stay and 30-day readmission rate were not significantly different between patient three groups (*p* = 0.17 vs *p* = 0.36) although patients with POI in vedolizumab group showed significantly longer length of postoperative stay compared to those without POI in vedolizumab group ((9 (6–12)) vs 5 (3–5)); *p* = 0.004)).

**Table 3:** Surgical Outcomes of the Three Study Cohorts with Case-Matched

	None (*n* = 54)	Anti-TNF (*n* = 74)	Vedolizumab (*n* = 25)	p
Overall morbidity	20 (37)	33 (45)	11 (44)	0.67
Infectious complications	11 (20)	8 (11)	2 (8)	0.20
*Pelvic abscess*	0	2 (3)	1 (4)	
*Wound infection*	9 (17)	5 (8)	1 (4)	
*Urinary tract*	1 (2)	0	0	
*Pneumonia*	1 (2)	0	0	
Post-operative Ileus				
*Grade I*	5 (9)	12 (16)	9 (36)	0.01
*Grade II*	3(6)	4(5)	2(8)	0.89
Other morbidity	4 (8)	13 (18)	1(4)	0.14
Postoperative stay (d)	6 (5–8)	6 (5–8)	5 (3–9)	0.17
30-day readmission	7 (13)	11 (15)	1 (4)	0.36

All values expressed as median (range) or n (%)

*TNF* tumor necrosis factor

Clinical factors associated with the development of Grade I-POI are shown in Table 4. Univariate analysis with total cohorts revealed that open surgery(*p* = 0.04), acute disease activity(*p* = 0.04), vedolizumab use significantly (*p* = 0.04) increased the incidence of POI. Interestingly, 6MP or MTX treatment had the opposite effect, significantly reducing the incidence of postoperative ileus (*p* = 0.05). Multivariable analysis confirmed that vedolizumab treatment prior to surgery was an independent risk factor for Grade I-POI (OR: 4.86 95% CI; 1.65–14.39; *p* = 0.004). Also, anti-TNF doubled the risk of Grade I-POI in multivariate analysis (OR: 2.45 95% CI;1.51–14.70; *p* = 0.01).

**Table 4:** Regression Analysis for Grade I- Postoperative Grade I Ileus with total cohort

	Univariate analysis			Multivariate analysis		
	OR	OR 95% CI	p	OR	OR 95%CI	p
Gender (M/F)	1.77	0.97–3.1	0.06			
Age at surgery(years)	1.01	0.94–1.02	0.94			
BMI (kg/m^2^)	0.11	0.41–2.7	0.55			
Preoperative wt loss (> 10 lb)^a^	1.05	0.41–2.7	0.92			
Disease duration (mos)	0.99	0.99–1.02	0.59			
Smoking						
*Never*						
*Prior*	0.97	0.47–2.0	0.97			
*Current*	0.67	0.79–5.7	0.72			
Disease extent						
*E2 Left-sided colitis*						
*E3 Pancolitis*	1.45	066–3.40	0.34			
Disease severity						
*S2 Moderate UC*						
*S3 Severe UC*	1.12	0.60–2.12	0.72			
Disease activity						
*Chronic*						
*Acute*	2.83	1.05–7.67	0.04	3.09	1.11–8.61	0.03
Medication history						
*Steroids*	0.92	0.59–1.98	0.92			
*6-MP or MTX*	0.53	0.29–0.97	0.04	0.56	0.28–1.07	0.08
IPAA stages						
*Total abdominal colectomy* *+end stoma*						
*Protectomy + IPAA with ileostomy*	1.24	0.65–2.40	0.51			
Operation type						
*Open*						
*laparoscopy*	0.53	0.28–0.98	0.04	0.54	0.27–1.06	0.07
Preoperative labs						
*Leukocyte (×10*^*3*^*/uL)*	1.00	1.00–1.00	0.45			
*Platelet (×10*^*3*^*/uL)*	1.01	0.99–1.00	0.34			
*Albumin (g/dL)*	0.82	0.40–1.65	0.58			
Biologic agent						
*None*						
*Anti-TNF*	1.76	0.83–3.73	0.14	2.45	1.08–5.59	0.03
*Vedolizumab*	3.15	1.08–9.2	0.04	4.71	1.51–14.70	0.01

*OR* odds ratio, *CI* confidence interval, *BMI* body mass index, *6-MP* 6-mercaptopurine, *MTX* methotrexate, *CSA* cyclosporine A, *IPAA* ileal pouch-anal anastomosis, *TNF* tumor necrosis factor

## Discussion

The literature regarding the effect on vedolizumab on adverse postoperative outcomes in IBD patients remains controversial. We therefore sought to understand outcomes following abdominal surgery for UC in those patients treated preoperatively with vedolizumab as compared to anti-TNF or no biologic therapy. We found the preoperative exposure to vedolizumab did not increase the rates of overall postoperative complications, infectious complications, or 30-day readmission compared to patients using anti-TNFs or those with no biologic drug exposure. However, we did find a novel association of preoperative vedolizumab exposure and the development of POI.

As with any immunosuppressive drug, surgeons have expressed concern regarding the potential for serious postoperative complications with the preoperative use of biologic drugs. There is considerable controversy regarding the potential adverse effects of anti-TNFs on surgical outcomes with studies showing conflicting results [12]. Factors responsible for this surgical controversy, such as retrospective study design, inclusion of both CD and UC patients, varying surgical procedures, and diverse patient populations have also accounted for reports showing inconsistent effects of vedolizumab on surgical outcomes. To address these issues, our study prospectively collected surgical outcomes in only UC patients undergoing one of two procedures. We further minimized study bias by case matching vedolizumab exposed, anti-TNF exposed and no biologic exposed patients. These methods resulted in patient cohorts well matched for clinical features, serum laboratory values, and operative procedures.

The current study adds to the 3 prior reports of vedolizumab in UC [8, 15, 16]. While one study showed a significant association with surgical site infection, our study concurs with the other two studies showing no deleterious effect of vedolizumab on overall complications and specifically infectious complications. In our whole cohort, patients with non-biologics tend to show high incidence of infection, but it would be related with high steroid use or higher incidence of IPAA. Except the infection complication rate, the total cohort showed similar tendency with 1:3 matching case control data of postoperative complication such as POI, postoperative stay or 30-day readmission rate.

A novel finding in our study was the significant association of preoperative vedolizumab use and the development of POI. While we acknowledge the lack of a consistent definition of POI in the literature [17], our definition of POI was the same across all 3 patient groups. Although POI is significantly higher in Vedolizumab group, postoperative stay did not show significant different between the 3 patient groups. Our data is long-term collected data, with recent treatment being early ambulation, early feeding to reduce ileus, and the length of hospital day being much shorter than before. Although the median values of the three groups do not seem to be statistically different, it was significantly longer in vedolizumab treated patients who developed POI compared with vedolizumab treated patients not developed POI (8.7 days vs 4.7 days). In addition, despite the numerous efforts to reduce POI with strictly followed ERAS protocol, it is more noteworthy that the incidence of ileus was highly developed in patients with the recently used medication, Vedolizumab. As we know, the α4β7 is focused on blocking leukocytes targeting the mucosa of the bowel while the anastomosis mainly heals within the serosa. So, we found the reason, not with wound healing but with bowel motility, for the effect on the master cell of α4β7. It is plausible but unproven that vedolizumab may influence mast cell stability with the subsequent development of POI. Mast cells are known to be important players in the intestinal inflammation that mediates dysfunction of gastrointestinal motility, and recent experimental studies have shown that mast cells have a high level of α4b7 integrin on their surface and strong contact of MAdcam-1 on other surfaces [18–20].

Another novel finding was the protective effect of 6-MP or MTX on the development of POI. All previous reports investigating the association of these immunomodulatory agents with increased postoperative complication rates in patients with IBD have been retrospective, with the two largest series focused on infectious complications without even mentioning POI in the analysis [21, 22]. While animal studies have demonstrated the prokinetic effects of MTX [23], we await future studies on the mechanism of immunosuppressive agents on gut motility in IBD to validate our findings with a larger number of patients.

The most glaring limitation of our study is the small number of vedolizumab treated patients with pre-exposure with anti-TNF, reflecting the relatively short period the drug has been approved for UC [24] combined with our center’s concern regarding the clinical utility of the drug’s slow onset of action in these severe UC patients. And, this study included the patients who have been treated since 2007 although we attempted to lower the bias by case-matching. This was due to the need for comparison with the non-biologic group, but it is possible that the surgical and medical therapies have changed between 2007 and 2014. We also recognize this model would not be suitable for all surgeons or all hospital, since this study was done in tertiary hospital by single surgeon. Difference hospital can have a significant difference in preoperative or postoperative treatment. Nevertheless, as our data show, Vedolizumab group increased the incidence of Grade I POI by four-fold compared with the non-biologic group and by 2 two-fold compared with the anti-TNF group, although all patients were treated with the same pre/post-operative management following ERAS protocol (A clear liquid diet continued < 2 h before general anesthesia; Mechanical bowel preparation plus oral antibiotic bowel preparation was done; Opioid-sparing pain control; Anti-emetic prophylaxis were added; Maintenance infusion of crystalloids should be tailored to avoid excess fluid administration; Nasogastric tube were avoided; Early and progressive patient mobilization; Early feeding< 24 h). Hence, we acknowledge that further validation of our findings with a larger number of patients is necessary. Nonetheless, our findings have shed further light into the UC patients who needed surgery prior to exposure of vedolizumab.

## Conclusions

Vedolizumab treated UC patients appear to be at risk for the development of POI. We encourage investigators to validate this finding in their own patient cohorts. In the interim, strategies for management of POI such as minimally invasive surgical technique, avoidance of a routine postoperative nasogastric tube, and use of opioid-sparing analgesia combined with early mobilization and oral feeding should be emphasized. There may also be a role for prokinetic agents such as alvimopan or methylnaltrexone [25–28].

## Data Availability

Not applicable.

## Abbreviations

6-MP: 6-mercaptopurine
AC: Abdominal colectomy
anti-TNF: Anti-tumor necrosis factor
BMI: Body mass index
CD: Crohn’s disease
ERAS: Enhanced Recovery After surgery
IBD: Inflammatory bowel disease
IC: Indeterminate colitis
IPAA: Ileal pouch-anal anastomosis
MAdCAM-1: Mucosal addressin cell adhesion molecule-1
MTX: Methotrexate
POI: Postoperative ileus
SBO: Small bowel obstruction
UC: Ulcerative colitis
